# Associations between Demographics, Tinnitus Specific-, Audiological-, General- and Mental Health Factors, and the Impact of Tinnitus on Daily Life

**DOI:** 10.3390/jcm11154590

**Published:** 2022-08-05

**Authors:** Maaike M. Rademaker, Inge Stegeman, Anne E. M. Brabers, Judith D. de Jong, Robert J. Stokroos, Adriana L. Smit

**Affiliations:** 1 Department of Otorhinolaryngology and Head and Neck Surgery, University Medical Center Utrecht, 3584 CX Utrecht, The Netherlands; 2 UMC Utrecht Brain Center, Utrecht University, 3584 CX Utrecht, The Netherlands; 3 Department of Ophthalmology, University Medical Center Utrecht, 3584 CX Utrecht, The Netherlands; 4 Epidemiology and Data Science, Amsterdam University Medical Centers, University of Amsterdam, 1012 WX Amsterdam, The Netherlands; 5 Nivel-Netherlands Institute for Health Services Research, 3513 CR Utrecht, The Netherlands; 6 Care and Public Health Research Institute, Maastricht University, 6211 LK Maastricht, The Netherlands

**Keywords:** tinnitus, tinnitus impact, TFI, associations, risk factors, tinnitus distress

## Abstract

Our objective was to study associations between demographics, tinnitus specific-, audiological-, general- and mental health characteristics, and impact of tinnitus in the general population. In this cross-sectional survey study in the Dutch population, data were prospectively gathered. Tinnitus impact was assessed with the Tinnitus Functional Index (TFI). We included participants who experienced tinnitus and for whom a total TFI score could be calculated (*n* = 212). We performed univariable and multivariable regression analyses. Due to logarithmical transformation, the B-scores were back-transformed to show the actual difference in points on the TFI. People who considered hyperacusis a small problem had a 12.5-point higher TFI score, those who considered it a mediocre problem had a 17.6-point higher TFI score and those who considered it a large problem had a 24.1-point higher TFI score compared to people who did not consider hyperacusis a problem. People who indicated having minor hearing problems had a 10.5-point higher TFI score, those with mediocre hearing problems had a 20.4-point higher TFI score and those with severe hearing problems had a 41.6-point higher TFI score compared to people who did not have subjective hearing problems. In conclusion, audiological risk factors, such as hearing problems and hyperacusis, have the largest association with the impact of tinnitus on daily life, compared to other assessed variables. The results of this study can be used in future research to find targeted interventions to diminish the impact of tinnitus.

## 1. Introduction

Tinnitus is a heterogeneous condition with considerable variations in onset, associated comorbidities and experienced impact on daily life [1]. Previously, McCormack et al. described a prevalence of tinnitus ranging between 5.1% and 42.7% in a systematic review [2]. However, not all individuals with tinnitus experience a negative effect on their daily life because of their tinnitus. Recently, those who suffer from its impact were defined as having a tinnitus disorder [3].

In their systematic review, Deklerk et al. described studies that assessed risk factors for tinnitus presence. They described numerous risk factors in different domains, including cardiovascular, psychological and neurological risk factors [4].

Factors associated with a larger experienced impact of tinnitus have also been described in various domains. Stress and mental diseases, such as anxiety or depression, have been described as psychological risk factors [5,6,7]. Moreover, somatic factors, such as hearing loss, or tinnitus specific risk factors, such as tinnitus loudness, have also been associated with tinnitus impact [6,7,8]. Nonetheless, the various studies on associations or risk factors and tinnitus impact have been performed in selected samples of patients [5,6,7,9], particularly studies focusing on patients seeking help for tinnitus. However, not all individuals who experience tinnitus seek help [10].

Detailed information about the associations between patient- and tinnitus-related characteristics and the impact of tinnitus on daily life could be of interest. Because not all people with tinnitus attend a healthcare provider, information about individuals with tinnitus among the general population is needed. This information can be used as a basis to design preventive strategies. Secondly, this could facilitate the identification of tinnitus subtypes in order to stratify individual treatment pathways. Therefore, in this study, we aim to provide insight into the associations between demographics, tinnitus characteristics, audiological-, mental- and general health factors, and tinnitus impact in a random sample of the general population.

## 2. Materials and Methods

This paper was written according to the Strengthening the Reporting of Observational Studies in Epidemiology (STROBE) statement [11]. 

### 2.1. Study Aim and Design

In this cross-sectional study, we aimed to assess risk factors with respect to the impact of tinnitus on daily life in a sample of the general population. Data were prospectively gathered with a postal or online survey (depending on the preference of the panel member) in January–February 2020. We sent the postal survey on 14 January 2020, and one postal reminder was sent on 30 January 2020. The online survey was sent on 16 January 2020, with two online reminders on the 23 January 2020 and 30 January 2020. The final date to fill out the survey was 14 February 2020. The survey was sent to members of the Nivel Dutch Health Care Consumer panel [12]. This panel was founded to measure, at a national level, opinions on and knowledge about health care, as well as expectations of and experiences with health care [13].

The Consumer Panel is a so-called ‘access panel’. An access panel consists of a large number of persons who have agreed to answer questions on a regular basis. In addition, many background characteristics of these persons (for example age, level of education, income and self-reported general health) are known. From the access panel, samples can be drawn for separate surveys. It is not possible for people to sign up on their own initiative. The panel is renewed on regular base. Renewal is necessary to make sure that members do not develop specific knowledge of and attention to healthcare issues and that no ‘questionnaire fatigue’ occurs. Moreover, renewal compensates for panel members who, for example, have died or moved without informing the panel of their new address [13].

This study is part of a larger study designed to describe tinnitus prevalence, tinnitus characteristics and healthcare usage. The sample for this larger study included panel members (*n* = 2251) who allowed for linkage of their survey answers with healthcare consumption data as registered by their general practitioner [14]. We recently published two studies on the same database [10,15]. The complete survey can be found in the appendix of Rademaker et al. [10]. For the current study, we included only data of participants with tinnitus for whom a total score of the impact of tinnitus on daily life measured by the Tinnitus Functional Index (TFI) could be calculated [16].

### 2.2. Outcome

#### The Impact of Tinnitus on Daily Life 

As part of the survey, the impact of tinnitus on daily life was assessed with the multi-item TFI questionnaire [16]. Participants were asked to answer the TFI questions if they were defined as having tinnitus based on the frequency and duration of the experienced tinnitus, as previously described by Rademaker et al. [10]. To this end, people were classified as having tinnitus when they experienced tinnitus for 5–60 min (daily or almost daily or weekly) or >60 min or continuously (daily or almost daily or weekly or monthly).

25 questions on a 11-point Likert scale, make up the TFI. The final score alters between 0 and 100 [16]. A score between 0 and 17 can be interpreted as not a problem, 18–31 as a small problem, 32–53 as a moderate problem, 54–72 as a big problem and 73–100 as a very big problem [16,17]. The 25 questions of the TFI are a combination of scores of impact on daily life out of eight subcategories (each subcategory is measured with 3 to 4 questions): intrusiveness, sense of control, cognition, sleep, hearing, relaxation, quality of life and emotions. The TFI was developed and validated in the USA, Tromp et al. translated it from English to Dutch and validated the translation in 2014. The Dutch translation exhibits high internal consistency (Cronbach’s alpha of 0.91 [16,17,18]).

### 2.3. Variables

The choice of variables to be addressed was based on known risk factors for tinnitus impact reported in the literature and based on expert opinion. Please see Supplementary Method S2 of Rademaker et al. for the exact wording of the questions and answer options with respect to the categorical variables [10].

#### 2.3.1. Tinnitus Specific Variables

The following items were assessed as tinnitus specific variables: being a help seeker (defined as when participant had either sought help for tinnitus or planned to seek help (yes/no)), tinnitus pattern (continuous/intermittent), subjective problem of tinnitus (no problem/small problem/moderate problem/large problem/very large problem), when did the tinnitus start (<3 months ago, 3–6 months ago, ≥6 months ago), whether the tinnitus varied in loudness (yes/no) and the tinnitus pitch (high/average/low/I don’t know).

#### 2.3.2. General Health Variables

The following general health item was assessed: subjective presence of chronic pain (yes/no).

#### 2.3.3. Mental Health Variables

Symptoms of anxiety and depression were measured with the Hospital Anxiety and Depression Scale (HADS) [19,20]. This is a 14-item questionnaire that uses a four-point scale to measure symptoms of anxiety (HADS-A; seven items) and depression (HADS-D; seven items). The HADS was translated to Dutch and validated (Cronbach’s alpha 0.71 and 0.90 for HADS-A, HADS-D and total scale). The total scores for the anxiety and depression scales range from 0 to 21. A score of ≥ eight indicates a possible depression or anxiety [20].

#### 2.3.4. Audiological Variables

We used the questions about whether sounds were a problem (no problem/small problem/mediocre problem/large problem/very large problem), hereafter referred to as hyperacusis, and the presence of hearing problems (no problems/small problems/mediocre problems/severe problems/I hear nothing) as audiological variables.

#### 2.3.5. Demographic Variables

The following items were assessed as demographic variables: age (at date of questionnaire submission), gender and level of education (low/middle/high). These were gathered when participants joined the panel and were provided by Nivel for this study.

### 2.4. Data Handling and Ethics

Data were analyzed anonymously, and the privacy of the panel members is guaranteed, as is described in the privacy policy of the Dutch Health Care Consumer Panel [13]. This complies with the General Data Protection Regulation (GDPR). According to Dutch legislation, it is not obligatory to obtain informed consent or approval from a medical ethics committee for research conducted through the panel (CCMO, 2020). The Medical Research Ethics Committee (MREC) of the University Medical Center Utrecht (UMC Utrecht) confirmed on 20 November 2019, that the Medical Research Involving Human Subjects Act (WMO) does not apply to this study and that therefore, official approval of the MREC is not required under the Human Subjects Act (MREC local protocol number 19–745). This study was performed according to the Declaration of Helsinki.

### 2.5. Statistical Analysis

Statistical analyses were performed with SPSS version 25.0.0.2. [21]. Normality of variables was visually assessed. Frequencies, means, standard deviation (SD), medians and interquartile ranges (IQR) were calculated for the variables of the total study group. To assess the relative importance of the characteristics to the TFI score, both univariable linear regression analyses and multivariable linear regression analyses were performed (complete case). The following patient characteristics were assessed: gender, age, level of education, tinnitus pattern, subjective problem of tinnitus, start of tinnitus, varying loudness, tinnitus pitch, being tinnitus help seeker, having chronic pain, HADS-A and HADS-D score, presence of hyperacusis and hearing problems. Based on expert opinion and literature reports, in a second analysis, we adjusted for the following potential confounders: gender, age and presence of hearing loss. Multivariable analyses were performed for all above-mentioned variables to assess their effect on TFI score (except for the single-item score of the subjective problem of tinnitus, as this outcome resembles the multi-item TFI score). The risk factors of gender, age and presence of hearing loss were each corrected for the other two potential confounders. To satisfy the assumption of normal distribution of residuals, the TFI was logarithmically transformed. Afterwards, residuals were approximately normally distributed. All other assumptions were satisfied. The outcomes were presented as B (95% CI) of this logarithmic scale and back-transformed to show the actual difference in points on the TFI scale according to each variable. Categorical variables were dummy-coded. A *p* value of 0.05 or lower was considered statistically significant.

## 3. Results

The survey was sent to 2251 panel members. Nine hundred and thirty-two (41.4%) panel members filled out the questionnaire. Out of these 932 respondents, 216 (23.2%) participants were classified as a tinnitus participant based on the stated definition. We were able to calculate the total TFI for 212 of 216 participants (98.1%, 4 missing); therefore, 212 participants were included in this study.

The mean age of the 212 participants was 66.2 (SD 10.8) years. A total of 122 of 212 (57.5%) were male. Among the participants, 135 (63.7%) had a continuous pattern of tinnitus, compared to 77 (36.3%) who had an intermittent pattern. The loudness of the tinnitus varied for 105 (49.5%) of the participants. A total of 72 of 212 (34.0%) participants were defined as help seekers. A total of 35 of 212 (16.5%) of the participants experienced chronic pain. Furthermore, 78 (36.8%) of 212 did not experience any hearing problems, whereas 70 (33.0%) experienced small hearing problems, 47 (22.2%) experienced mediocre problems, 13 (6.1%) experienced severe problems and 4 (1.9%) experienced complete hearing loss (Answer option: “yes, I hear nothing”) (Table 1).

### 3.1. Outcomes of Univariable and Adjusted Analysis with Respect to TFI Outcomes

#### 3.1.1. Demographic Variables

Age or gender were not statistically significantly associated with TFI score in the univariable and adjusted analyses (age: univariable: B = −0.003 (95% CI −0.014–0.009), *p* = 0.664, adjusted: −0.01 (−0.02–0.00), *p* = 0.49/gender: univariable (male = reference) female B = 0.19 (95% CI −0.7–0.44), *p* = 0.15, adjusted B = 0.18 (95% CI −0.07–0.43), *p* = 0.16) (Table 1).

#### 3.1.2. Tinnitus Specific Variables

Participants with a continuous tinnitus pattern had a significantly higher TFI score in the univariable and adjusted regression analyses than those with an intermittent tinnitus pattern (univariable, B = −0.52 (95% CI −0.77–0.27), *p* = 0.000, adjusted: B = −0.45 (95% CI −0.70–−0.20), *p* = 0.000). When back-transformed, this resulted in an 8.1-point higher score on the TFI for a continuous pattern compared to an intermittent pattern in the univariable analysis and a 11.1-point higher score in the adjusted analysis (Table 1).

In the univariable analysis, the score for the question about experiencing problems with having tinnitus (scale 1 to 5; no problem to very large problem) was associated with a higher TFI. When back-transformed, we found that the answer option ‘’no problem’’ corresponded to a TFI score of 6.5, ‘’small problem’’ to a score of 16.2, ‘’moderate problem’’ to a score of 31.3, ‘’large problem’’ to a score of 55.9 and ‘’very large problem’’ to a score of 73.9.

Individuals with a varying loudness of tinnitus had a significantly higher TFI score than those with non-varying tinnitus loudness (univariate B = 0.45 (95% CI 0.19–0.70) *p* = 0.000, adjusted: 0.36 (0.10–0.61) *p* = 0.006). This resulted in a 7.5-point higher TFI score for a varying loudness compared to a non-varying loudness in the univariate analysis and a 7.4-point difference in the adjusted analysis.

#### 3.1.3. General Health Variable

Having chronic pain was associated with a higher TFI than not experiencing chronic pain in univariable analysis (B = 0.46 (95% CI 0.13–0.79), *p* = 0.007), as well as in the adjusted analyses (B = 0.44 (95% CI 0.12–0.76), *p* = 0.008). This resulted in an 8.9-point higher score on the TFI for participants with chronic pain compared to those without chronic pain in the univariable analyses and 12.9 in the adjusted analyses.

#### 3.1.4. Mental Health Variables

In the univariable and adjusted analyses, both the HADS-A and the HADS-D were associated with a higher TFI score ((HADS-A univariable B = 0.12 (95% CI 0.09–0.15), *p* = 0.000), HADS-D B = 0.10 (95% CI 0.07–0.13), *p* = 0.000/adjusted HADS-A B = 0.11 (95% CI 0.08–0.14), *p* = 0.000, HADS-D B = 0.09 (95% CI 0.06–0.12), *p* = 0.000). Based on the adjusted analyses, this resulted in a TFI score of 11.8 in those with a median HADS-A score (3.0). If an individual’s HADS-A score increased by one (to 4.0), it would result in a TFI score of 13.2. For the HADS-D, the median score was one. The TFI score was 13.1 for those with a median HADS-D score (1.0) and 14.2 for those with an increase in the median HADS-D score of one (2.0).

#### 3.1.5. Audiological Variables

Having hyperacusis was associated with a higher TFI score in univariable and adjusted analyses (small problem: univariable B = 0.50 (95% CI 0.19–0.80), *p* = 0.001 (back-transformed TFI = 20.5), adjusted B = 0.44 (95% CI 0.14–0.75), *p* = 0.005 (back-transformed TFI = 35.2), mediocre problem: univariable B = 0.71 (95% CI 0.39–1.03), *p* = 0.000 (back-transformed TFI = 25.4), adjusted B = 0.58 (95% CI 0.23–0.92), *p* = 0.001 (back-transformed TFI = 40.3) and large problem: univariable B = 0.99 (95% CI 0.36–1.62), *p* = 0.002 (back-transformed TFI = 33.5), adjusted B = 0.72 (95% CI 0.07–1.38), *p* = 0.031 (back-transformed TFI = 46.8).

Hearing problems were associated with significantly higher TFI scores in both the univariable and adjusted analyses (small problem: univariable B = 0.36 (95% CI 0.07–0.65), *p* = 0.02 (back-transformed TFI = 17.5), adjusted B = 0.36 (95% CI 0.07–0.7), *p* = 0.016 (back-transformed TFI = 34.7), mediocre problems: univariable B = 0.49 (95% CI 0.16–0.82), *p* = 0.003 (back-transformed TFI = 20.0), adjusted B = 0.61 (95% CI 0.27–0.95), *p* = 0.001 (back-transformed TFI = 44.6), severe problems: univariable B = 0.82 (95% CI 0.29–1.35), *p* = 0.003 (back-transformed TFI = 27.8), adjusted B = 1.00 (95% CI 0.45–1.55), *p* = 0.000 (back-transformed TFI = 65.8), I hear nothing: univariable B = 1.06 (95% CI 0.16–1.97), *p* = 0.02 (back-transformed TFI = 35.5), adjusted B = 1.14 (95% CI 0.22–2.05), *p* = 0.015 (back-transformed TFI = 75.7).

## 4. Discussion

In this cross-sectional study of a general population sample, we assessed whether several demographic-, tinnitus specific-, general- and mental health characteristics were associated with the impact of tinnitus on daily life as measured with the TFI.

We included different domains of variables in our survey. Audiological factors were most important compared to the other assessed risk factors in terms of association with tinnitus impact. Tinnitus specific characteristics seemed to be less important, which is in line with the results of a study by Beukes et al. [9]. In this cross-sectional study in a hospital population, the authors concluded that tinnitus-related comorbidities were more strongly associated with tinnitus impact in comparison to demographic variables (including tinnitus specific factors) [9].

When back-transformed, the variables of hearing loss, hyperacusis and chronic pain had a difference of more than 13 points on the TFI between two answer options in the multivariable analyses. A 13-point difference in TFI score is considered to be the minimal clinically important difference to be perceived as an effect or change [17]. Therefore, these factors can potentially make a difference in terms of an individual’s experienced impact of tinnitus on daily life according to on our study results. These three factors have also been identified as risk factors in other studies [9,22,23,24].

Anxiety and depression are commonly described to be associated with tinnitus [4]. In our study, the association found between HADS-A and HADS-D and the TFI was relatively small. This might be explained by several reasons, such as the nature of the sample (general population) or by the fact that these measures only scored symptoms instead of having an anxiety or a depressive disorder itself. In addition, we did not correct for any potential treatment or medications for anxiety or depression that might have altered anxiety or depressive symptoms and therefore the observed association.

The outcome of the single question, “how big of a problem is your tinnitus”, with a scoring in five categories, was found to be associated with the scales of impact defined for the TFI score [16,17]. Specifically, in three out of five categories, this single-item score was very close or within the cut-off values of the originally defined TFI scales [17]. For example, the answer option, ‘large problem’ on the single-item question predicted a TFI score of 55.9 points, which falls within the range of the defined TFI scale ‘large problem’ (TFI score 54–72). Currently, lengthy questionnaires are used to assess tinnitus impact. The results of our study could be of interest for population studies wherein tinnitus prevalence and impact are assessed [2]. Rather than having to administer a lengthily questionnaire, a single-item question may suffice.

The major strength of the current study is the assessment of associations with tinnitus impact in a non-clinical sample. Nonetheless, certain limitations are applicable to our study. First, although we invited a sample of the Dutch population to participate in our survey, the response resulted in a sample of participants with a higher mean age than is representative of the overall Dutch population [25]. Secondly, in survey research, there is always a balance between the urge to ask more questions and the limitations of the length of the questionnaire in terms of burden on the participant. The already-lengthy questionnaire might have been of consequence to the limited response rate (41.4%). We only asked participants who experienced tinnitus sounds of a certain frequency and duration to fill out the TFI to measure the impact of tinnitus on daily life [10,15]. In hindsight, it would have been interesting to assess the impact of the experienced tinnitus more broadly. Consequently, we might have missed several participants who did not meet our criteria of being a tinnitus participant but in whom tinnitus might have affected their daily life.

How can the outcomes of our study be used in clinical care and future research? The associations we found highlight the effect of comorbidities on tinnitus impact, not only in those who seek help but also in those in the general population. Based on the present study, we cannot draw any conclusions about the causality or mechanisms of the found associations, nor about the appropriateness of findings fitting one of the current pathophysiological models of tinnitus [26,27,28]. However, in clinical care, it might be helpful to ask patients about the studied associations with tinnitus impact. Future preventive measures for tinnitus impact might be targeted at the associations found in this study and could be targeted toward these groups. However, whether therapy or preventive measures that focus on common risk factors actually diminishes the impact of tinnitus on daily life remains to be determined by further research.

## 5. Conclusions

In this study, we assessed associations between demographics, tinnitus specific-, audiological-, general- and mental health factors, and the impact of tinnitus on daily life, as measured with the TFI. Based on the ultimate effect on the TFI score of the different variables, we can conclude that audiological variables, such as hearing problems and hyperacusis, have the largest effects on the TFI compared to the other variables assessed.

## Figures and Tables

**Table 1:** Baseline characteristics, Univariable and Multivariable B-scores and back transformed TFI scores.

		N (%)	Median TFI Score	Univariable (B (95% CI))	Univariable Back-Transformed	Multivariable ^1^ (B (95% CI))	Multivariable Back-Transformed
**Demographic**							
Gender	Male	122 (57.5)	14.2 (22.8)	Ref		Ref	
	Female	90 (42.5)	20.2 (20.2)	0.19 (−0.07–0.44)		0.18 (−0.07–0.43)	
	Missing/constant	0 (0)		2.72)		3.19	
Age ^1^ (n = 212)	Median (IQR)	66 (15)		−0.003 (−0.01–0.01)		−0.01 (−0.02–0.00)	
	Constant			2.97		3.19	
Education	Low	27 (12.7)	30.4 (46.1)	0.47 (0.08–0.86) *	23.8	0.45 (0.06–0.84) *	38.5
	Middle	74 (34.9)	15.0 (20.0)	0.06 (−0.21–0.34)		0.07 (−0.20–0.34)	
	High	104 (49.1)	14.8 (21.7)	Ref	14.9	Ref	24.5
	Missing/constant	7 (3.3)		2.70		3.20	
**Tinnitus specific**							
Pattern	Continuous	135 (63.7)	20.0 (25.2)	Ref	19.9	Ref	30.6
	Intermittent	77 (36.3)	11.6 (16.2)	−0.52 (−0.77–−0.27) *	11.8	−0.45 (−0.70–−0.20) *	19.5
	Missing/constant	0 (0.0)		2.99		3.42	
Subjective problem	No problem	50 (23.6)	7.4 (6.1)	Ref	6.5		
	Small problem	103 (48.6)	16.4 (15.6)	0.91 (0.68–1.14) *	16.2		
	Moderate problem	43 (20.3)	40.4 (33.6)	1.57 (1.29–1.84) *	31.3		
	Large problem	12 (5.7)	60.2 (11.1)	2.15 (1.72–2.57) *	55.9		
	Very large problem	4 (1.9)	73.6 (11.3)	2.43 (1.74–3.12) *	73.9		
	Missing/constant	0 (0.0)		1.87			
When did it start?	<3 months ago	7 (3.3)	8.8 (23.6)	−0.44 (−1.14–0.26)		−0.62 (−1.31–0.06)	
	3−6 months ago	9 (4.2)	16.4 (25.7)	0.15 (−0.47–0.77)		0.22 (−0.38–0.81)	
	≥6 months	196 (92.5)	17.7 (21.5)	Ref		Ref	
	Missing/constant	0 (0.0)		2.81		3.29	
Varying loudness	No	99 (46.7)	14.0 (20.4)	Ref	13.1	Ref	17.3
	Yes	105 (49.5)	21.6 (27.4)	0.45 (0.20–0.70) *	20.6	0.36 (0.10–0.61) *	24.7
	Missing/constant	8 (3.8)		2.57		2.85	
Tinnitus pitch	High	75 (35.4)	14.8 (22.0)	Ref		Ref	
	Average	73 (34.4)	15.6 (22.8)	0.04 (−0.26–0.34)		0.02 (−0.28–0.32)	
	Low	42 (19.8)	20.6 (28.3)	0.22 (−0.13–0.58)		0.21 (−0.14–0.56)	
	I don’t know	16 (7.5)	21.6 (18.6)	0.08 (−0.43–0.58)		0.01 (−0.49–0.51)	
	Missing/constant	6 (2.8)		2.74		3.27	
Tinnitus help seeker	No	140 (66.0)	14.7 (19.1)	Ref	13.7	Ref	21.5
	Yes	72 (34.0)	22.8 (43.1)	0.55 (0.94–0.81) *	23.8	0.47 (0.21–0.73) *	34.3
	Missing/constant			2.62		3.07	
**General health**							
Chronic pain	No	177 (83.5)	14.8 (19.0)	Ref	15.3	Ref	23.5
	Yes	35 (16.5)	31.6 (36.0)	0.46 (0.13–0.79) *	24.2	0.44 (0.12–0.76) *	36.4
	Missing/constant	0 (0.0)		2.73		3.16	
**Mental health**							
HADS-A ^1^ (n = 207)	Median (IQR)		3.0 (5.0)	0.12 (0.09–0.15) *		0.11 (0.08–0.14) *	
	Constant			2.34		2.14	
HADS-D ^1^ (n = 209)	Median (IQR)		1.0 (5.0)	0.10 (0.07–0.13) *		0.09 (0.06–0.12) *	
	Constant			2.47		2.48	
**Audiological**							
Hyperacusis	No, no problem	120 (56.6)	12.4 (15.3)	Ref	12.5	Ref	22.7
	Yes, small problem	45 (21.2)	20.4 (28.0)	0.50 (0.19–0.80) *	20.5	0.44 (0.14–0.75) *	35.2
	Yes, mediocre problem	37 (17.5)	30.0 (41.8)	0.71 (0.39–1.03) *	25.4	0.58 (0.23–0.92) *	40.3
	Yes, large problem	8 (3.8)	53.6 (56.8)	0.99 (0.36–1.62) *	33.5	0.72 (0.07–1.38) *	46.8
	Yes, very large problem	1 (0.5)	21.1 **	0.53 (−1.21–2.26)	NS	−0.18 (−2.30–1.95)	NS
	Missing/constant	1 (0.5)		2.53		3.12	
Hearing problem	No, no problems	78 (36.8)	12.4 (14.9)	Ref	12.2	Ref	24.2
	Yes, small problems	70 (33.3)	19.8 (22.1)	0.36 (0.07–0.65) *	17.5	0.36 (0.07–0.7) *	34.7
	Yes, mediocre problems	47 (22.2)	20.8 (35.2)	0.49 (0.16–0.82) *	19.99	0.61 (0.27–0.95) *	44.6
	Yes, severe problems	13 (6.1)	47.6 (51.0)	0.82 (0.29–1.35) *	27.8	1.00 (0.45–1.55) *	65.8
	Yes, I hear nothing	4 (1.9)	39.0 (47.3)	1.06 (0.16–1.97) *	35.5	1.14 (0.22–2.05) *	75.7
	Missing/constant	0 (0.0)		2.51		3.19	

* Statistically significant, *p* < 0.05; ** no IQR presented because n < 4. Ref = reference. The back-transformed TFI score was calculated with the reference value as zero in the case of a statistically significant association. ^1^ Corrected for age, gender and hearing problems. The variable age, gender and hearing problems themselves were corrected for the other two problems (e.g., age was corrected for gender and hearing problems). For continuous risk factors, the back-transformed TFI score was based on the median score of the risk factor.

## Data Availability

The datasets presented in this article are not readily available because the Dutch Health Care Consumer Panel has a program committee, which supervises processing of the data of the Dutch Health Care Consumer Panel and decides about the use of the data. This program committee consists of representatives of the Dutch Ministry of Health, Welfare and Sport, the Health Care Inspectorate, Zorgverzekeraars Nederland (Association of Health Care Insurers in the Netherlands), the National Health Care Institute, the Federation of Patients and Consumer Organisations in the Netherlands, the Dutch Healthcare Authority and the Dutch Consumers Association. All research conducted within the Consumer Panel has to be approved by this program committee. The committee assesses whether a specific research project fits within the aim of the Consumer Panel, which is strengthened by the position of the healthcare user. Requests to access the datasets should be directed to the corresponding author.
